# Supplementary material for: A Likelihood Approach for Real-Time Calibration of Stochastic Compartmental Epidemic Models
Source: PLoS Comput Biol. 2017 Jan 17;13(1):e1005257. doi: 10.1371/journal.pcbi.1005257 (PMC5240920; doi:10.1371/journal.pcbi.1005257)
Supplement: S1 File — (TAR.GZ) [file pcbi.1005257.s014.tar.gz › HSPH_Online-SI-Revision/EnKF07/n10-severe/n10-severe_PF2_EnKF-summary2.pdf]

peak -8	3w spec	3w cum	5w spec	5w cum	end	3w spec LOG	3w cum LOG	5w spec LOG	5w cum LOG	end LOG
mean	433.9	1379.1	91.1	365.9	67.1	0.9	0.8	1.5	0.6	0.6
median	182.5	818.	89.8	284.6	61.6	0.8	0.8	1.4	0.6	0.4
quantile .1	82.3	85.4	66.8	74.1	50.1	0.4	0.3	0.7	0.4	0.3
quantile .9	703.2	3533.3	107.	856.5	87.7	1.3	1.4	2.2	0.4	1.2
40	15	21	24	35						

peak -4	3w spec	3w cum	5w spec	5w cum	end	3w spec LOG	3w cum LOG	5w spec LOG	5w cum LOG	end LOG
mean	89.6	102.9	92.5	84.9	80.8	1.3	0.7	2.	0.8	0.9
median	90.6	92.2	93.3	84.3	85.9	1.4	0.7	2.3	0.8	1.
quantile .1	67.3	76.3	86.9	66.	59.	0.6	0.3	1.1	0.3	0.4
quantile .9	105.5	141.2	97.2	103.5	91.2	2.1	0.9	2.6	0.3	1.3
11	33	0	24	19						

peak	3w spec	3w cum	5w spec	5w cum	end	3w spec LOG	3w cum LOG	5w spec LOG	5w cum LOG	end LOG
mean	98.1	103.9	99.3	99.1	95.	1.4	0.8	1.8	0.8	0.9
median	94.2	99.	97.2	98.5	94.6	1.3	0.8	1.8	0.8	0.8
quantile .1	85.1	80.6	88.3	80.7	80.	0.9	0.5	1.2	0.6	0.7
quantile .9	110.6	132.6	110.7	118.1	110.5	2.1	1.	2.7	0.6	1.1
13	30	3	30	30						

peak +4	3w spec	3w cum	5w spec	5w cum	end	3w spec LOG	3w cum LOG	5w spec LOG	5w cum LOG	end LOG
mean	139.4	163.3	189.5	152.9	158.2	1.	0.5	1.2	0.5	0.5
median	116.5	154.7	112.2	134.6	128.9	0.8	0.5	1.1	0.5	0.5
quantile .1	81.8	76.4	79.5	81.	93.9	0.5	0.3	0.7	0.3	0.3
quantile .9	213.	267.3	309.4	253.7	244.7	1.7	0.6	1.8	0.3	0.7
38	45	37	47	47						

peak +8	3w spec	3w cum	5w spec	5w cum	end	3w spec LOG	3w cum LOG	5w spec LOG	5w cum LOG	end LOG
mean	12 698.	3686.2	9550.3	3870.5	4298.4	1.1	0.7	1.4	0.7	0.7
median	398.1	727.	318.4	717.7	641.3	1.	0.6	1.3	0.6	0.6
quantile .1	103.6	229.7	92.9	209.1	157.3	0.5	0.4	0.6	0.3	0.4
quantile .9	11 292.5	4292.1	38 726.	4391.1	4367.9	1.9	1.1	2.	0.3	1.2
43	26	36	28	28	{}	{}	{}	{}	{}	{}
